# Identification and validation of a QTL for spikelet number on chromosome arm 6BL of common wheat (*Triticum aestivum* L.)

**DOI:** 10.1007/s11032-022-01288-7

**Published:** 2022-03-21

**Authors:** Andrew Katz, Patrick Byrne, Scott Reid, Sarah Bratschun, Scott Haley, Stephen Pearce

**Affiliations:** Department of Soil and Crop Sciences, Colorado State University, Fort Collins, CO 80523 USA

**Keywords:** Spikelet number, Kernel weight, Heading date, Heterogeneous Inbred Family, Wheat

## Abstract

To provide food security for a growing world population, it will be necessary to increase yields of staple crops such as wheat (*Triticum aestivum* L.). Yield is a complex, polygenic trait influenced by grain weight and number, which are negatively correlated with one another. Spikelet number is an important determinant of grain number, but allelic variants impacting its expression are often associated with heading date, constraining their use in wheat germplasm that must be adapted for specific environments. Identification and characterization of genetic variants affecting spikelet number will increase selection efficiency through their deployment in breeding programs. In this study, a quantitative trait locus (QTL) on chromosome arm 6BL for spikelet number was identified and validated using an association mapping panel, a recombinant inbred line population, and seven derived heterogeneous inbred families. The superior allele, *QSn.csu-6Bb*, was associated with an increase of 0.248 to 0.808 spikelets per spike across multiple environments that varied for mean spikelet number. Despite epistatic interactions between *QSn.csu-6B* and three other loci (*WAPO-A1*, *VRN-D3*, and *PPD-B1*), genotypes with a greater number of superior alleles at these loci consistently exhibit higher spikelet number. The frequency of superior alleles at these loci varies among winter wheat varieties adapted to different latitudes of the US Great Plains, revealing opportunities for breeders to select for increased spikelet number using simple molecular markers. This work lays the foundation for the positional cloning of the genetic variant underlying the *QSn.csu-6B* QTL to strengthen our understanding of spikelet number determination in wheat.

**Supplementary Information:**

The online version contains supplementary material available at 10.1007/s11032-022-01288-7.

## Introduction

Common wheat (*Triticum aestivum* L.) provides 20% of global caloric intake and comprises a quarter of cereal production by volume (FAOSTAT). Therefore, it is imperative to improve wheat yields to meet the consumption needs of a growing population (Ray et al. 2013). Wheat yield is a complex, polygenic trait determined by both grain weight and grain number per unit area (Fischer 1985), which are negatively correlated with one another as a result of resource competition (Shanahan et al. 1985; Borrás et al. 2004; Zhang et al. 2016; Mangini et al. 2018). A reductionist approach to study the genetic variation underlying individual yield components contributing to overall yield has been proposed to overcome this complexity (Brinton and Uauy 2019). Identification and characterization of genetic variants influencing yield components will improve our understanding of the epistatic interactions between different alleles, and allow breeders to more precisely select optimal allelic combinations to develop higher-yielding wheat cultivars.

Wheat yield is most closely associated with grain number m^−2^ (Kuchel et al. 2007; Bennett et al. 2012; Xie and Sparkes 2021), which itself is determined by tiller number, spikelet number per spike, and spikelet fertility (Slafer et al. 1990, 2015). The wheat spike is located at the terminus of each productive tiller and is composed of spikelets formed at each rachis node, each with the potential to form between four and six grains (Bonnett 1966). Spikelet number is determined early in reproductive development (Bonnett 1966), has a high broad-sense heritability observed between 0.49 and 0.95 (Zhang et al. 2018; Ma et al. 2019; Chen et al. 2020), and is less affected by the environmental factors that impact grain number and weight later in the growing season (Zhang et al. 2018). Therefore, breeding for increased spikelet number is a promising approach to develop higher-yielding wheat cultivars (Borrás et al. 2004; Slafer et al. 2015; Wolde et al. 2019).

Wheat plants with a winter growth habit initiate the transition from vegetative to reproductive development following vernalization and in response to lengthening photoperiods (Hyles et al. 2020). Optimizing the timing of this transition for each target environment is crucial to maximize a wheat plant’s reproductive success, which in agriculture translates to grain production. Plants that initiate spike development too early risk exposure to late-spring frost damage, while heading too late increases the risk of heat stress during grain filling (Kamran et al. 2014; Grogan et al. 2016). Allelic variation in several genes has been identified that influences both heading date and spike architecture, creating a challenge for wheat breeders to increase spikelet number without delaying heading date beyond the optimal environmental window. For example, *PHOTOPERIOD 1* (*PPD1*) encodes a PSEUDO RESPONSE REGULATOR family protein that accelerates heading in response to long-day photoperiods (Beales et al. 2007). Plants with tandem duplications of the *PPD-B1* gene (*Ppd-B1a* allele) exhibit reduced sensitivity to photoperiod and head earlier than plants with fewer copies (*Ppd-B1b* allele (Díaz et al. 2012)). The *Ppd-B1b* allele is associated with increased spikelet number in durum wheat (*Triticum turgidum* L. var. *durum*) (Arjona et al. 2018) and regulates spikelet formation (Boden et al. 2015).

*PPD1* accelerates heading in part through the activation of *VERNALIZATION 3* (*VRN3*, also called *TaFT1*), which encodes an ortholog of *Arabidopsis thaliana FLOWERING LOCUS T1* (Yan et al. 2006). In *A. thaliana* and rice (*Oryza sativa*), *FT1* encodes a mobile protein that is translocated from the leaves to the shoot apical meristem to induce meristem identity genes that accelerate heading date and promote reproductive development (Corbesier et al. 2007; Tamaki et al. 2007). A single nucleotide insertion in the *Vrn-D3b* allele creates a frame shift mutation conferring a later heading date compared to plants carrying the *Vrn-D3a* allele (Bonnin et al. 2008; Wang et al. 2009; Chen et al. 2010). The later heading date conferred by a homoeologous *Vrn-B3b* allele (referred to as *TaFT-B1a*) is associated with increased spikelet number and is proposed to be caused by a non-synonymous SNP (Brassac et al. 2021). Allelic variation in *PPD1* and *VRN3* genes has been broadly utilized in wheat breeding programs to optimize heading date for specific target environments (Nishida et al. 2013; Zheng et al. 2013; Grogan et al. 2016; Ochagavía et al. 2018).

Other quantitative trait loci (QTL) for spike architecture have less impact on heading date. Through positional cloning, *WHEAT ORTHOLOG OF ABERRANT PANICLE ORGANIZATION 1* (*WAPO-A1*) was identified as a candidate gene for a spikelet number QTL on chromosome arm 7AL (Kuzay et al. 2019). *WAPO-A1* is an ortholog of *A. thaliana UNUSUAL FLORAL ORGANS* (Wilkinson and Haughn 1995) and rice *ABERRANT PANICLE ORGANIZATION 1*, and overexpression of this gene confers increased spikelet number in rice (Ikeda et al. 2005, 2007). In wheat, the *Wapo-A1b* allele is associated with increased spikelet number in some environments (Kuzay et al. 2019; Muqaddasi et al. 2019) and its frequency in wheat germplasm has increased over time, potentially as a consequence of positive selection in formal breeding programs (Kuzay et al. 2019). Although the mechanism by which *WAPO-A1* affects spikelet number remains unknown, natural variation in *WAPO-A1* has a limited effect on heading date (Muqaddasi et al. 2019).

Another QTL associated with grain weight and spike architecture was previously identified on wheat chromosome arm 6BL and is the focus of the current research study. Using doubled haploid and recombinant inbred line (RIL) populations derived from two hard white winter wheat genotypes (“Platte” and CO940610), associations were detected between this QTL and test weight (El-Feki et al. 2013; Dao et al. 2017), kernel weight and diameter (Dao et al. 2017), and spike length (El-Feki et al. 2018). The superior allele for test weight and kernel weight from the genotype CO940610 was also associated with higher grain protein and ash concentration in a segregating BC_3_F_2:3_ population (Dao et al. 2017).

In the current study, high-density genetic markers from the wheat 90 K SNP array (Wang et al. 2014) and exome capture and sequencing technologies (Krasileva et al. 2017) were used to validate and characterize this QTL, which was associated with spikelet number in different genetic populations. This genetic locus was thus named *QSn.csu-6B*, where *QSn.csu-6Ba* was associated with wider grains and fewer spikelets and *QSn.csu-6Bb* was associated with narrower grains and more spikelets. Although the *QSn.csu-6B* locus exhibits epistatic interactions with previously characterized *WAPO-A1*, *VRN-D3*, and *PPD-B1* alleles for spikelet number, genotypes with greater numbers of superior alleles at these loci consistently exhibit higher spikelet number. Geographic variation in the frequencies of each allele among germplasm reveals opportunities to apply marker assisted selection to combine superior alleles for this trait in wheat breeding programs.

## Materials and methods

### Plant materials

The Hard Winter Wheat Association Mapping Panel (HWWAMP) is a population of 258 hard red and 41 hard white winter common wheat inbred lines assembled by public and private breeding programs reflecting the wide precipitation and temperature gradients across the primarily dryland wheat cropping systems of the Great Plains region of the USA (Guttieri et al. 2015). The lines were previously categorized into northern (Montana, North Dakota, South Dakota, *n* = 39), central (Colorado, Kansas, Nebraska, *n* = 119), and southern (Oklahoma, Texas, *n* = 105) classes, based on the geographical region within the Great Plains where the lines were developed (Grogan et al. 2016). A fourth category of “other” (*n* = 36) was used for lines that did not fit one of these three regional categories.

The CO940610/ “Platte” hard white winter wheat recombinant inbred line (COP-RIL) population was previously described (Dao et al. 2017). Remnant F_5:6_ seed from this population was advanced to produce F_5:7_ and F_5:8_ seed used in field experiments. CO940610 (GSTR 10702; pedigree — KS87H22/MW09) is an experimental hard white winter wheat line developed by the Colorado State University wheat breeding program and carries *Ppd-B1b*, *Vrn-D3a*, *Wapo-A1a*, and *QSn.csu-6Ba* alleles. “Platte” (PI 596297; pedigree — “Tesia 79”/Chat “S”// “Abilene”) is a hard white winter wheat cultivar developed by HybriTech Seed International and carries *Ppd-B1a*, *Vrn-D3b*, *Wapo-A1b*, and *QSn.csu-6Bb* alleles.

Eight Heterogeneous Inbred Families (HIFs) were derived from COP-RIL F_3:4_ remnant seed by screening 528 individual plants with Kompetitve Allele Specific PCR (KASP) marker BobWhite_c22638_135 (Table S1). The 35 individual plants heterozygous for this marker were genotyped with three other KASP markers (KS0619_760354, KS0619_760357, and KS0619_760427) spanning the *QSn.csu-6B* locus. The high background genomic homogeneity in near isogenic lines developed from these individuals can provide greater power to detect allelic effects in mapping studies (Haley et al. 1994; Tuinstra et al. 1997). Individuals heterozygous for all four loci were then genotyped using KASP markers to distinguish *WAPO-A1*, *VRN-D3*, and *PPD-B1* alleles (Table S1). From the 35 individuals, eight HIFs at the F_4:5_ generation were derived, each with a different combination of fixed *PPD-B1*, *WAPO-A1*, and *VRN-D3* alleles (Table S2). From each HIF, 96 individual F_5_ plants were genotyped for the *QSn.csu-6B* locus with the same four KASP markers to identify single plants fixed for either the *QSn.csu-6Ba* or *QSn.csu-6Bb* allele. F_5:6_ seeds were derived from these individuals and used in field experiments.

### Field experiments

Field experiments for the HWWAMP were planted in Greeley, Colorado (40.447 N, 104.636 W; elevation 1425 m; soil type Nunn clay loam and Olney fine sandy loam) under irrigated and dryland conditions in the fall of 2011, referred to as “Greeley 2012 irrigated” and “Greeley 2012 dryland,” respectively. The population was planted again in Fort Collins, Colorado (40.648 N, 104.993 W; elevation 1558 m; soil type Nunn clay loam) under irrigated and dryland conditions in the fall of 2012, referred to as “Fort Collins 2013 irrigated” and “Fort Collins 2013 dryland,” respectively. Plots approximately 3.6 m^2^ were arranged in a row-column design with two check varieties “Settler CL” (PI 655,242; pedigree — “Wesley” sib// “Millennium” sib/ “Above” sib) and “Hatcher” (PI 638,512; pedigree — “Yuma”/PI 372,129// “TAM-200”/3/4*Yuma/4/KS91H184/ “Vista”). The experimental genotypes were unreplicated for each environment.

Field experiments for the biparental populations were planted in Fort Collins, Colorado. The F_5:6_, F_5:7_, and F_5:8_ COP-RIL populations were planted under irrigated conditions in the fall of 2016, 2018, and 2019, referred to as “Fort Collins 2017,” “Fort Collins 2019,” and “Fort Collins 2020,” respectively. Each line was planted in a two-row plot 0.92 m long with 23 cm spacing between rows, 28 cm spacing between plots, and a planting density of approximately 2,500,000 seeds ha^−1^. In Fort Collins 2017, two replicates were planted in a randomized complete block design. In Fort Collins 2019 and Fort Collins 2020, one replicate was planted where the RILs were randomized. Eight F_5:6_ HIFs were planted in Fort Collins 2020 under the same conditions as the RILs. Each HIF was planted in three replicates using a randomized complete block design with a family block nested within each replicate block. Each replicate block consisted of all eight family blocks, and each family block consisted of 18 lines with nine lines fixed for *QSn.csu-6Ba* and nine lines fixed for *QSn.csu-6Bb*.

### Phenotyping

Grain weight, heading date, and spikelet number data were previously collected for the HWWAMP in four environments: Greeley 2012 dryland, Greeley 2012 irrigated, Fort Collins 2013 dryland, and Fort Collins 2013 irrigated (Grogan et al. 2016). These phenotypic data were downloaded from the T3/Wheat database (Blake et al. 2016). Kernel width was measured for the current study from remnant seed of the HWWAMP grown in the same four environments using the method described below.

Grain length, width, and weight were measured for the COP-RIL population in Fort Collins 2017. Each sample consisted of ten spikes randomly selected from each plot. The grain was threshed using a wheat head thresher (Precision Machine Co., Inc, Lincoln, NE, USA, part # WHTA0100001). Broken grains were discarded, and approximately 400 grains were spread evenly across a bed scanner (Microtek International Inc, Hsinchu City, Taiwan, part # MRS-3200A3L). Scanned images were collected at a vertical and horizontal resolution of 300 dots per inch and analyzed using the software GrainScan (Whan et al. 2014) to calculate mean length, width, and grain number. Sample weight was divided by grain number to calculate mean grain weight.

Spikelet number was measured in each environment by manually counting spikelets beginning from the first rachis node to the terminal spikelet. Only heads with an intact terminal and basal spikelet were counted. For Fort Collins 2017, ten spikes were randomly selected from each plot, and grains harvested from those spikes were used to measure grain length, width, and weight. Twenty spikes were selected for Fort Collins 2019 and Fort Collins 2020.

Heading date was recorded in Julian days from January 1 when approximately 50% of the spikes in a plot had fully emerged from the flag leaf sheath. Heading date was recorded for each line of the COP-RIL and HIF populations in Fort Collins 2020. All raw phenotypic data is provided in Supplemental data 1.

### Genotyping

Lines from the HWWAMP population were previously genotyped using the 90 K SNP array (Wang et al. 2014), and data were downloaded from the T3/Wheat database (Blake et al. 2016). Markers with a minor allele frequency of greater than 0.05 and missing in less than 20% of genotypes were retained for downstream analysis (Supplemental data 1). Peak markers BobWhite_c22638_135 and IWA5913 from the 90 K SNP array data were used to differentiate between the *QSn.csu-6B* and *WAPO-A1* alleles, respectively. Marker data for *PPD-B1* was generated using a PCR-based assay (Beales et al. 2007) and were described for the HWWAMP previously (Grogan et al. 2016). *VRN-D3* was genotyped using a cleaved amplified polymorphic sequence marker previously described (Chen et al. 2010).

DNA was extracted using a modified Cetyl Trimethyl Ammonium Bromide (CTAB) extraction protocol (Doyle and Doyle 1990). DNA samples were diluted in water to a final concentration between 50 and 100 ng/µL prior to genotyping. DNA concentration was measured via a spectrophotometer (Thermo Scientific, Waltham, MA, USA, NanoDrop 1000). KASP reactions were performed in a total volume of 10 µL comprising 5 µL KASP V4.0 2X Master Mix (LGC Genomics, LLC, Beverley, MA, USA, Cat. # KBS-1016–017-US), 0.14 µL of 54 µM KASP primer mix, and approximately 100 ng of DNA template. The PCR conditions used consisted of: 94 °C for 15 min for hot-start *Taq* activation; ten cycles of two-step PCR with a 94 °C denaturing step for 20 s and an annealing-elongation step for 60 s starting at 61 °C and decreasing by 0.6 °C each cycle until reaching 55 °C; 26 further cycles of two-step PCR with a 94 °C denaturing step for 20 s and a 55 °C annealing-elongation step for 60 s. PCR was carried out on an Applied Biosystems QuantStudio Q3 qPCR machine (Applied Biosystems, Waltham, MA, USA, Cat. # A28136) and analyzed using QuantStudio Design and Analysis software (version 1.4). KASP primer sequences for the *VRN-D3* locus were designed from a previously described cleaved amplified polymorphic sequence marker and provided by the US Department of Agriculture, Agricultural Research Service, Hard Winter Wheat Genetics Research Unit (Manhattan, KS, USA) (Table S1). KASP primer sequences for the *WAPO-A1* locus were previously published (Table S1). KASP primer sequences for the *QSn.csu-6B* alleles were designed using the online primer design tool PolyMarker (Ramirez-Gonzalez et al. 2015). The *PPD-B1* locus was genotyped using a presence-absence marker adapted from the previously described PCR-based assay by using the same KASP PCR conditions, but with 42 µM of KASP primer mix with only one forward primer including a VIC fluorophore tail sequence (Table S1) (Beales et al. 2007). Primers were synthesized by Integrated DNA Technologies (San Diego, CA, USA). All genotypic data is provided in Supplemental data 1.

### Statistical analysis

Grain width best linear unbiased estimates (BLUEs) were calculated for the HWWAMP with the lmer function of the afex R package version 0.28–1 (Singmann et al. 2020) using a mixed model where environment and the row-column location of each plot was treated as a random effect and genotype was treated as a fixed effect. The BLUEs model also accounted for the nested structure of plot location within each environment. Genome-wide association mapping was conducted upon 16,058 markers and 298 genotypes using a mixed model method previously described (Yu et al. 2006) and R version 4.0.2 (Team R Development Core 2020) with the rrBLUP version 4.6.1 package (Endelman 2011). In the mixed model, population structure was treated as a fixed effect using an additive relationship matrix calculated from the markers, and genotype was treated as a random effect. Marker effect was treated as a fixed effect following the restricted maximum likelihood method previously described (Endelman 2011). QTL were identified as significant markers (− Log10 (*P*) > 3) within 10.0 cM of one another, using the genetic map previously published for the 90 K SNP array (Supplemental data 1) (Wang et al. 2014).

Spikelet number, heading date, and kernel weight BLUEs for the HWWAMP were calculated with a linear model considering both genotype and environment as fixed effects due to data from the T3/Wheat database being corrected prior to this analysis. BLUEs calculated for the COP-RIL population used the same method; however, the Fort Collins 2017 data was averaged across two replicates prior to analysis to avoid confounding estimates due to the unbalanced nested structure of two replications in Fort Collins 2017; no replication was included in Fort Collins 2019 and Fort Collins 2020.

Spikelet number and kernel weight data for the HWWAMP were analyzed via one-way analysis of variance (ANOVA) using R version 4.0.2 (Team R Development Core 2020) with the package emmeans version 1.5.0 (Lenth 2019). Only 249 lines that had genotype and phenotype data for all four loci of interest (*WAPO-A1*, *VRN-D3*, *PPD-B1*, and *QSn.csu-6B*) and four environments (Greeley 2012 dryland, Greeley 2012 irrigated, Fort Collins 2013 dryland, and Fort Collins 2013 irrigated) were used to calculate the effect sizes and *P-*values.

Pearson correlations were estimated using the Pearson’s Product-Moment Correlation Test from the stats R package (R version 4.0.2, stats package version 4.0.2). HWWAMP correlations for each environment were calculated between spikelet number, heading date and kernel weight for each environment, and BLUEs across all environments. The Fort Collins 2017 COP-RIL correlations were calculated among kernel length, kernel width, kernel weight, and spikelet number. The Fort Collins 2020 COP-RIL correlations were calculated between heading date and spikelet number.

One-way ANOVA was conducted for all kernel length, kernel width, kernel weight, heading date, and spikelet number observations of the COP-RIL population with the loci of interest as the dependent variable using R version 4.0.2 (Team R Development Core 2020) with the package emmeans version 1.5.0 (Lenth 2019). Prior to analysis, COP-RIL individuals that were segregating at the *WAPO-A1*, *VRN-D3*, *PPD-B1*, and *QSn.csu-6B* loci were removed. To identify interactions affecting spikelet number, a full linear model was constructed accounting for the four-way interaction between the *WAPO-A1*, *VRN-D3*, *PPD-B1*, and *QSn.csu-6B* loci and environment. Akaike information criterion was calculated for all possible model subset combinations of the full model using the MuMin package in R (R version 4.0.2, MuMIn package version 1.43.17) (Barton 2020). The model with the lowest Akaike information criterion was the full four-way interaction model, which was used for subsequent analyses on interactions. Significance of the model terms was calculated via the Tukey-adjusted type II ANOVA method.

The HIF spikelet number data was analyzed via a linear model accounting for the allelic state of the *QSn.csu-6B* locus by family and the replicate effect using R version 4.0.2 with the package emmeans version 1.5.0. Since the *WAPO-A1*, *VRN-D3*, and *PPD-B1* alleles were nested within each family, these variables were not included in the model. Using the backwards selection approach, a model that accounted for the effect of the *QSn.csu-6B* locus, family, and replicate was identified, which was used for all downstream analysis.

## Results

### Identification and validation of a QTL for grain width and spikelet number in the HWWAMP

**Fig. 1:**
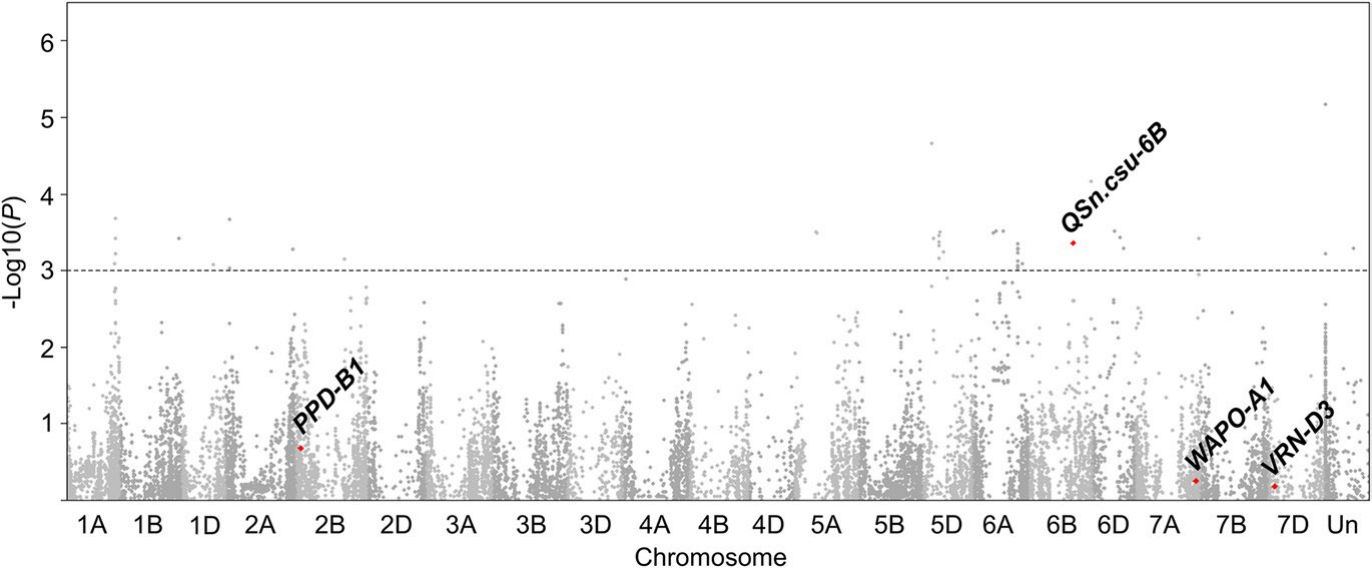
A genome-wide association of the hard winter wheat association mapping panel (HWWAMP) for kernel width using 16,058 markers derived from the 90 K SNP array (Wang et al. 2014). Best linear unbiased estimates for kernel width were calculated for 298 genotypes using data collected from the Greeley 2012 dryland, Greeley 2012 irrigated, Fort Collins 2013 dryland, and Fort Collins 2013 irrigated environments. The horizontal line represents − Log10 (*P*) = 3. The closest flanking markers for each locus of interest were determined by their physical position in the IWGSC RefSeq v1.0 genome assembly and are highlighted in red

A genome-wide association study of kernel width in the HWWAMP identified 46 significant marker associations for grain width (− Log10 (*P*) > 3) corresponding to 12 QTL (Fig. 1; Supplemental data 1). None of these QTL corresponded to the peak markers for *WAPO-A1*, *VRN-D3*, and *PPD-B1*, which all exhibited no association with variation in kernel width (− Log10 (*P*) = 0.25, 0.18, and 0.68, Table S3). One QTL, with a single significant marker (BobWhite_c22638_135, − Log10 (*P*) = 3.36) and two flanking markers, spanning a 10.0 Mbp region of chromosome arm 6BL in the RefSeq v1.0 reference genome assembly (493.5 Mbp to 503.5 Mbp; Supplemental data 1) was previously associated with variation in grain traits (El-Feki et al. 2013; Dao et al. 2017). This peak marker is more than 360 Mbp from the *GPC-B1* locus, ruling out *NAM-B1*, that is associated with variation in grain protein content, as a candidate gene (Uauy et al. 2006). To investigate this QTL further, the peak marker (BobWhite_c22638_135) was used to analyze the association with kernel weight and spikelet number using publicly available phenotypic data from 249 lines of the HWWAMP population grown in four environments (Table 1). Variation at the *QSn.csu-6B* locus was significantly associated with kernel weight in all four individual environments (*P* < 0.05), indicating that the increase in grain width conferred by *QSn.csu-6Ba* translates to heavier grains (Table 1). BLUEs across all four environments showed that the *QSn.csu-6Ba* allele is significantly associated with increased kernel weight (*P* = 0.0002). The *QSn.csu-6B* locus was also significantly associated with spikelet number in one environment (Greeley 2012 dryland, *P* = 0.02; Table 1), where the *QSn.csu-6Bb* allele conferred increased spikelet number.

**Table 1:** Effect size of *QSn.csu-6B*, *WAPO-A1*, *VRN-D3*, and *PPD-B1* loci on thousand kernel weight (TKW, in grams) and spikelet number per spike (SNS) in the HWWAMP across four environments and the calculated BLUEs across all environments. Significance was calculated via one-way ANOVA where *P < 0.05, **P < 0.01, ***P < 0.0001; n.s = not significant

Environment	*QSn.csu-6B*		*WAPO-A1*		*VRN-D3*		*PPD-B1*	
	TKW	SNS	TKW	SNS	TKW	SNS	TKW	SNS
Greeley 2012 dryland	1.30**	0.48*	0.15n.s	1.05***	1.02**	0.16n.s	0.07n.s	0.07n.s
Greeley 2012 irrigated	0.01*	0.05n.s	0.01n.s	1.21***	0.01**	0.52**	0.01**	0.33*
Fort Collins 2013 dryland	0.94**	0.11n.s	0.77**	0.73***	0.68**	0.56***	0.01n.s	0.06n.s
Fort Collins 2013 irrigated	1.82**	0.11n.s	0.43n.s	0.98***	1.10**	0.69***	0.11n.s	0.08n.s
BLUEs	1.02**	0.05n.s	0.34n.s	0.99***	0.70**	0.48***	0.05n.s	0.07n.s

Kernel weight and spikelet number were negatively correlated in three of the four tested environments (*P* < 0.001; Table S4) and based on BLUEs calculated from all four environments (*r* = − 0.260, *P* < 0.0001; Table S4). Although there was no significant correlation between kernel weight and spikelet number (*P* = 0.634) in the Greeley 2012 dryland environment, these traits showed the strongest negative correlation in the Greeley 2012 irrigated environment (*r* = − 0.408, *P* < 0.0001; Table S4).Among three other loci of interest that were tested, variation in *WAPO-A1* was significantly associated with spikelet number in all four environments (*P* < 0.0001; Table 1) and in a GWAS (− Log10 (*P*) = 7.64; Table S3). Calculated BLUEs showed that the *WAPO-A1b* allele is associated with increased spikelet number (*P* < 0.0001). Variation in *VRN-D3* was significantly associated with both kernel weight and spikelet number in all environments (*P* < 0.01), except spikelet number in Greeley 2012 dryland (*P* = 0.25). Consistent with the negative correlations between these traits, the *Vrn-D3b* allele was significantly associated with increased spikelet number (*P* < 0.0001) and the *Vrn-D3a* allele was significantly associated with increased kernel weight (*P* = 0.0003). Variation in *PPD-B1* was associated with spikelet number and kernel weight only in Greeley 2012 irrigated (*P* < 0.001; Table 1) and with heading date in a GWAS (− Log10 (*P*) = 3.55) (Table S3).

### Epistatic and additive interactions between four spikelet number loci in the COP-RIL population

**Fig. 2:**
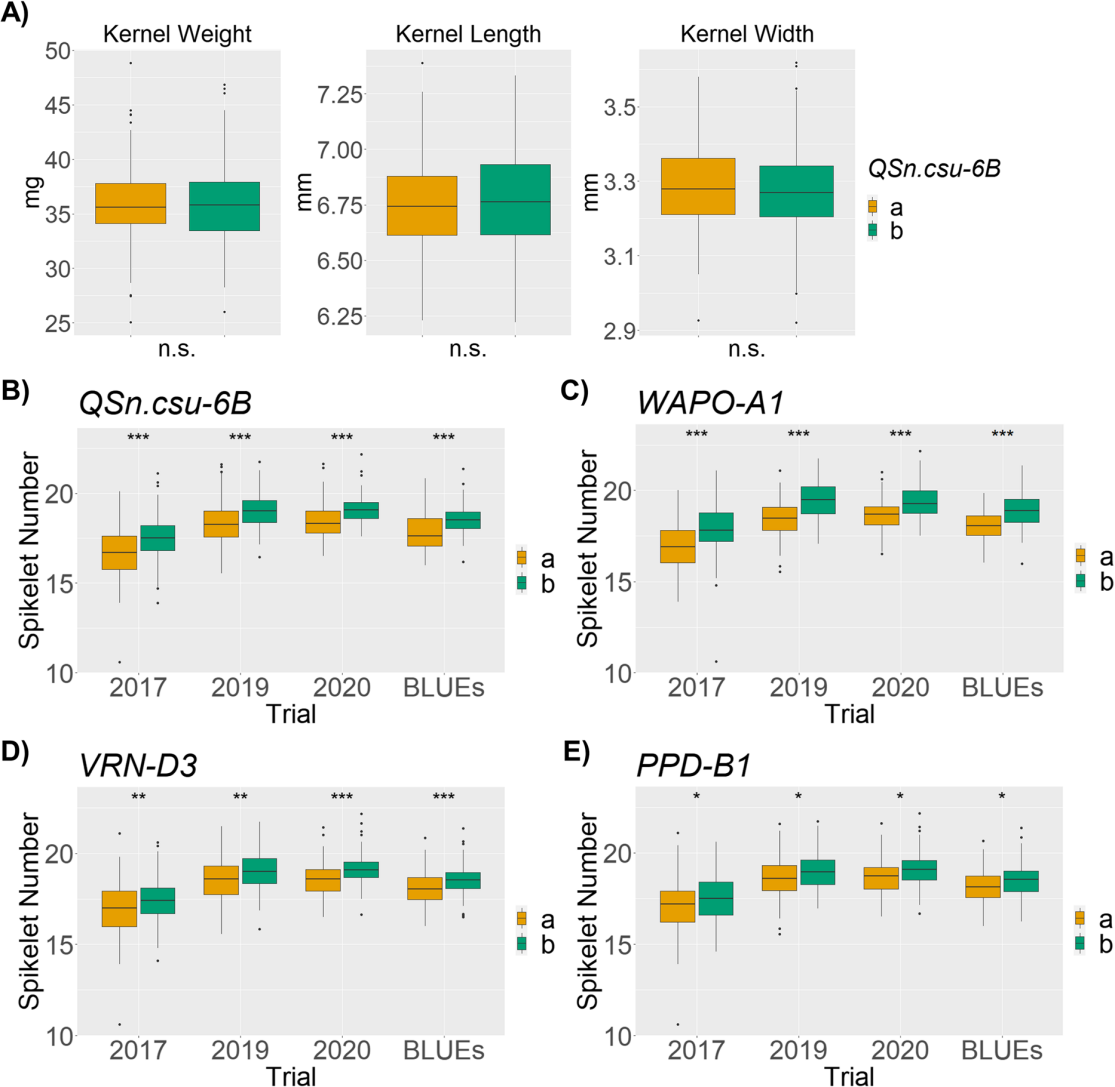
Marker-trait associations observed in the COP-RIL population in three environments. **A** Association between *QSn.csu-6B* alleles and kernel weight, length, and width in Fort Collins 2017. The effect of the “Platte” and CO940610 alleles on spikelet number for the **B** *QSn.csu-6B*, **C** *WAPO-A1*, **D** *VRN-D3*, and **E** *PPD-B1* loci in Fort Collins 2017, 2019, and 2020 environments and the Best Linear Unbiased Estimates (BLUEs). *P < 0.05, **P < 0.001, ***P < 0.0001, calculated via one-way ANOVA

The genotypes CO940610 and “Platte” are polymorphic for all four loci of interest, so the COP-RIL population was used to further validate their association with grain size and weight, and spikelet number in three environments. In Fort Collins 2017, there was no significant marker-trait association between *QSn.csu-6B* and grain weight, width or length (Fig. 2A). All three traits were negatively correlated with spikelet number (*P* < 0.05; Table S5). A significant marker-trait association was detected between *QSn.csu-6B* and spikelet number in the Fort Collins 2017, 2019, and 2020 environments (Fig. 2B). The *QSn.csu-6Bb* allele derived from “Platte” conferred a positive effect of between 0.679 and 0.808 spikelets among the three environments, explaining between 9.2 to 12.9% of the variation (Table S6). When BLUEs calculated from all three environments were analyzed, *QSn.csu-6B* had a significant effect of 0.739 spikelets (*P* < 0.0001; Table S6).

Variation in *WAPO-A1*, *PPD-B1*, and *VRN-D3* loci segregating in the COP-RIL population were also significantly associated (*P* < 0.05) with spikelet number in all three environments (Fig. 2C-E). For all three loci, the superior allele was consistently associated with increased spikelet number in all three environments (Table S6). The *WAPO-A1b* allele conferred the largest effect of 0.890 spikelets (*P* < 0.0001), consistent with the positive effect of 2.1 spikelets previously reported in a spring wheat RIL population (Kuzay et al. 2019). The *Vrn-D3b* allele had a positive effect of 0.518 spikelets (*P* < 0.0001), and the *Ppd-B1b* allele had a positive effect of 0.394 spikelets (*P* = 0.003), consistent with a previous study in spring durum wheat RIL populations (Arjona et al. 2018).

**Fig. 3:**
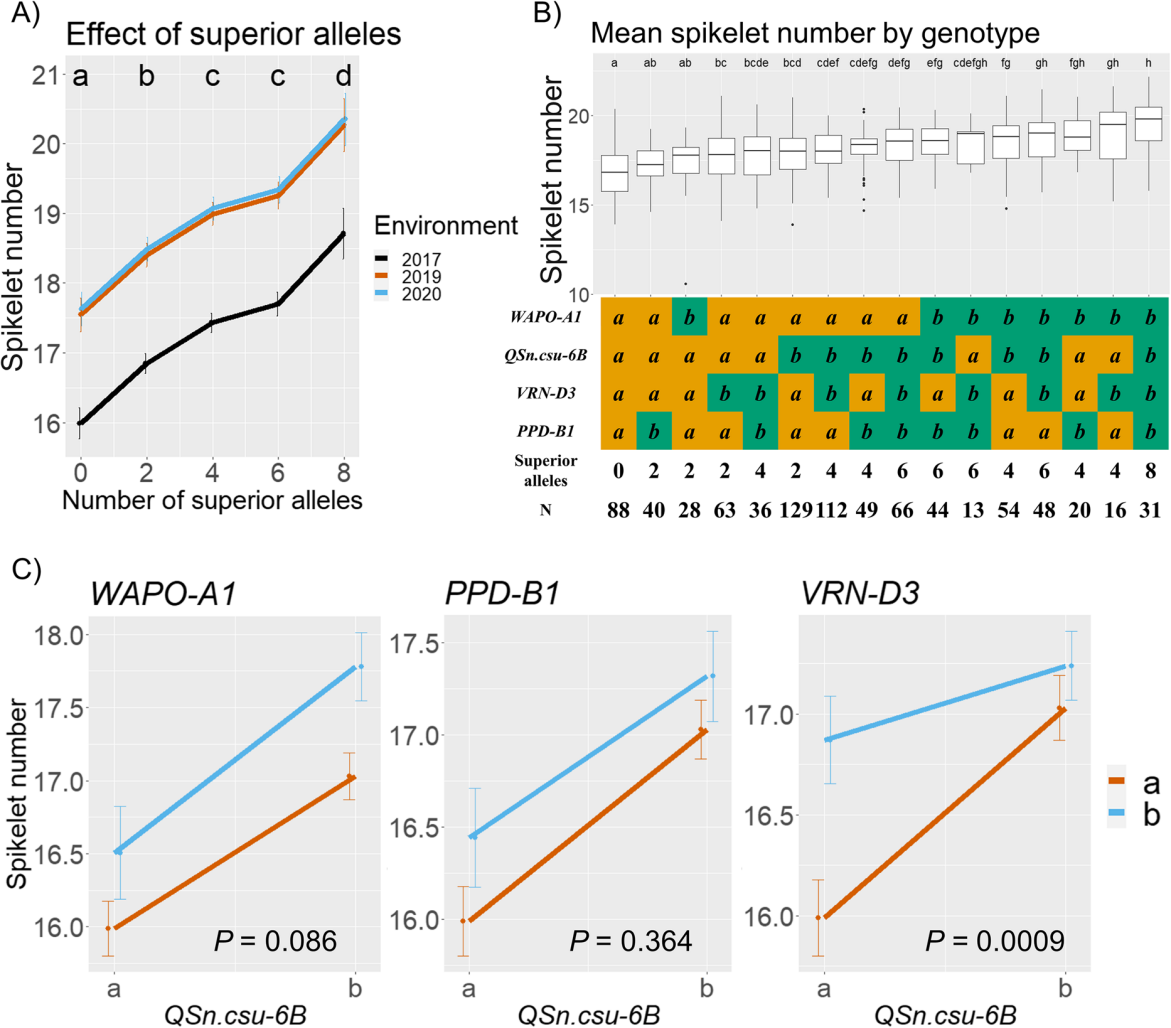
Effect of combining superior spikelet number alleles across four loci in the COP-RIL population. **A** Mean spikelet number for the Fort Collins 2017, 2019, and 2020 environments by number of superior alleles. Compact letter display represents significant differences between genotypes (*α* = 0.05). **B** Grand mean spikelet number for each allelic combination. Compact letter display represents significant differences between genotypes (*α* = 0.05). **C** *Q* graphs of two-way interactions between *QSn.csu-6B* and the three other loci

Genotypes that carried a greater number of superior alleles at these four loci consistently exhibited a higher spikelet number compared to genotypes with fewer superior alleles, even when the mean spikelet number within environments differed significantly (Fig. 3A; Table S7). Pairwise comparisons between genotypes grouped by their number of superior alleles show that spikelet number significantly increases as the number of superior alleles increase (*P* < 0.0001), except for the comparison between genotypes carrying two and three superior alleles (*P* = 0.0531; Fig. 3A). When genotypes were separated according to their specific allelic combinations at these four loci, the pattern was similar, but differences between genotypes were smaller (Fig. 3B). This may be a result of a significant two-way interaction between *QSn.csu-6B* and *VRN-D3* (*P* = 0.0009) (Fig. 3C), a three-way interaction among *QSn.csu-6B*, *VRN-D3*, and *PPD-B1* (*P* = 0.0002; Table S7) and a four-way interaction among all loci (*P* < 0.0001; Table S7). This is supported by an allelic frequency analysis where the *QSn.csu-6B* effect on spikelet number is greatest in genetic backgrounds with both *VRN-D3a* and *PPD-B1a* alleles (Fig. S1). Two-way interactions between *QSn.csu-6B* and *WAPO-A1* (*P* = 0.086) and between *QSn.csu-6B* and *PPD-B1* (*P* = 0.364) were not significant (Fig. 3C).

In Fort Collins 2020, spikelet number and heading date were positively correlated in the COP-RIL population (*r* = 0.37, CI95 = 0.25–0.48, *P* < 0.0001), consistent with previous observations (Shaw et al. 2013; Chen et al. 2020; Brassac et al. 2021). Among individual loci, variation in *PPD-B1* and *VRN-D3* were significantly associated with heading date (*P* < 0.0001; Table S8). Furthermore, the effect of *PPD-B1* and *VRN-D3* on spikelet number was not independent of heading date, suggesting the observed effect on spikelet number is a result of a difference in heading (Fig. S2). By contrast, variation at the *WAPO-A1* and *QSn.csu-6B* loci was not associated with heading date (Table S8) and the effect of these loci on spikelet number was independent of heading date (Fig. S2).

### Validation of *QSn.csu-6B* effect on spikelet number in HIFs

**Fig. 4:**
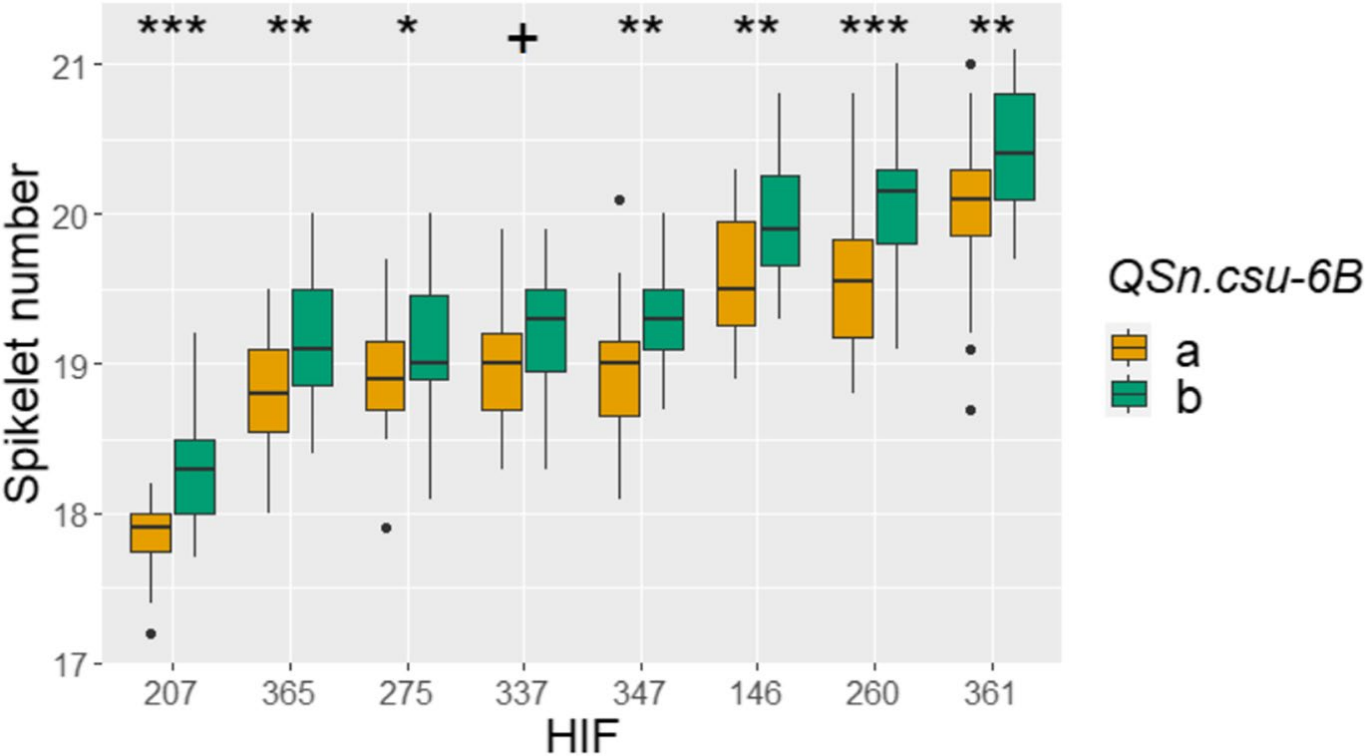
Mean spikelet number for each *QSn.csu-6B* allelic class in eight HIFs. + *P* < 0.1, *P < 0.05, **P < 0.01, ***P < 0.001. Full details of each HIF are provided in Table S2

The effect of *QSn.csu-6B* on spikelet number was further validated in eight HIFs segregating for this locus, but fixed for different combinations of alleles at *VRN-D3*, *PPD-B1*, and *WAPO-A1* (Table S2). The *QSn.csu-6B* locus was significantly associated with spikelet number in seven of the eight HIFs (*P* < 0.05), with an effect size ranging from 0.248 to 0.504 spikelets (Table S2). The positive effect of the *QSn.csu-6Bb* allele on spikelet number was consistent in all seven families despite variation between families in mean spikelet number (Fig. 4) and *VRN-D3*, *PPD-B1*, and *WAPO-A1* alleles (Table S2). For example, the largest effect sizes for the *QSn.csu-6B* allele were observed in families COP6BHF260 (mean spikelet number of 19.8, fixed for *Vrn-D3b* and *Ppd-B1b* alleles) and COP6BHF207 (mean spikelet number of 18.1, fixed for the *Ppd-B1b* allele; Table S2). The *QSn.csu-6Bb* allele was also associated with increased spikelet number in family COP6BHF337, although the difference was not significant (*P* = 0.0667). This family headed two days earlier than any other HIF and was fixed for the early flowering *Vrn-D3a* and *Ppd-B1a* alleles (Table S2).

### A latitudinal dissection of the frequency of superior spikelet number alleles in HWWAMP

**Fig. 5:**
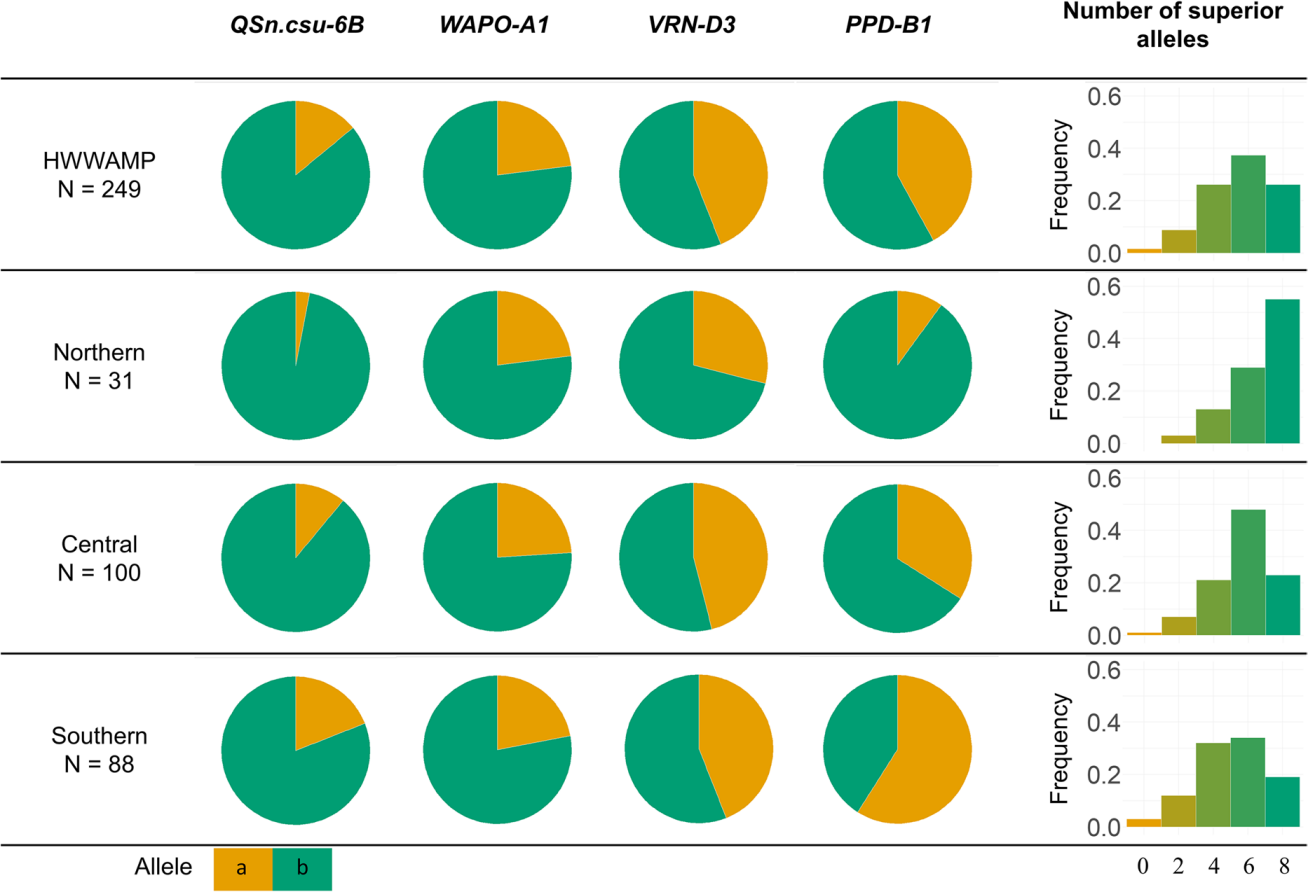
Allelic frequency of the superior (green) and inferior (orange) alleles for each locus affecting spikelet number (*QSn.csu-6B*, *WAPO-A1*, *VRN-D3*, and *PPD-B1)* across a latitudinal gradient within the HWWAMP. The top row contains all 249 genotypes, including 30 genotypes categorized as “other.” The right column represents the frequency of individuals which contain either 0, 2, 4, 6, or 8 superior alleles (*QSn.csu-6Bb*, *Wapo-A1b*, *Vrn-D3b*, and *Ppd-B1b*)

The frequency and distribution of superior spikelet number alleles for all four loci varied among germplasm from the HWWAMP adapted to different regions of the USA Great Plains (Fig. 5). The superior *QSn.csu-6Bb* allele had a frequency of 0.86 across the whole panel, but with increasing frequency in lines adapted to the northern region (0.97) compared to the southern region (0.81). The superior *Wapo-A1b* allele had a frequency of 0.77 among all lines, comparable to the frequency of this allele in a panel of North American spring wheat lines (0.83) (Kuzay et al. 2019), and varied only slightly between regions (Fig. 5). The superior *Vrn-D3b* allele was most common in lines adapted to the northern region (0.71), with lower frequencies in both the central (0.54) and southern (0.56) regions. The superior *Ppd-B1b* allele showed wide variation among regions, ranging from a frequency of 0.90 in the northern region to 0.41 in the southern region (Fig. 5). Across the entire HWWAMP 26% of lines carry all four superior alleles, while just 1.6% of lines carried no superior alleles at these loci (Fig. 5). The northern region had the largest proportion of lines carrying all four superior alleles (0.54) likely due to the overall greater frequency of superior *Ppd-B1b* and *Vrn-D3b* alleles (Fig. 5). A smaller proportion of lines with superior alleles at all four loci were observed in the central (0.23) and southern (0.19) regions.

## Discussion

### Application of the *QSn.csu-6B* QTL to increase spikelet number

In different bi-parental mapping populations tested in multiple environments, the *QSn.csu-6Bb* allele from “Platte” was consistently associated with increased spikelet number, despite this allele originally being identified for its association with reduced kernel weight in a doubled haploid population (El-Feki et al. 2013) and kernel width in an association mapping panel (Supplemental data 1). These observations are likely a factor of the well-documented negative correlation between spikelet number and grain weight (McIntyre et al. 2010; Bennett et al. 2012; Slafer et al. 2014), which was also observed in the current study (Table S5). However, despite this negative correlation in the COP-RIL population, *QSn.csu-6B* was associated only with variation in spikelet number (*P* < 0.001) and had no effect on kernel weight (*P* = 0.35). One possible explanation for these inconsistent results is that the QTL region includes more than one causal gene, with at least one gene impacting spikelet number and at least one gene controlling grain size. Depending on their proximity, the effects of both genes might be detected in a doubled haploid population or an association mapping panel as an overlapping QTL, whereas in the RIL population, it is possible that only the gene for spikelet number is segregating. High-resolution mapping to delimit the candidate gene region will be required to test this hypothesis. Environmental effects may also impact these observations, since the HWWAMP was evaluated in two environments with limited irrigation and two fully irrigated environments, including one year with above-average temperatures (Grogan et al. 2016). By contrast, the COP-RIL population was always evaluated in fully irrigated conditions, so differences in the expression of traits under these varying conditions could also be a factor in their inconsistency.

Although grain number is moderately correlated with yield *per se* (Kuchel et al. 2007; Bennett et al. 2012; Xie and Sparkes 2021), increased spikelet number does not always translate to greater grain number due to impacts at later stages of development, including on floret fertility (Bonnett 1966). Additionally, a skewed COP-RIL population structure favoring the positive effect allele *QSn.csu-6Bb* and negative effect alleles *WAPO-A1a*, *PPD-B1a*, and *VRN-D3a* (Table S9), could reduce the statistical power to detect the epistatic interactions identified in this study (Table S7). This could explain why *QSn.csu-6B* only had a significant effect on spikelet number in two of the eight allelic combinations (Fig. S1), whereas five of six allelic combinations were significant in the HIFs (Fig. 4; Table S2). Therefore, it will be important to evaluate the effects of the *QSn.csu-6B* locus using isogenic materials grown in replicated yield plots to determine the utility of this allele to breed for higher-yielding wheat varieties.

The effect of *QSn.csu-6B* on spikelet number is explained by a polygenic model with both additive and epistatic effects with *WAPO-A1*, *PPD-B1*, and *VRN-D3* alleles, and the inexpensive genetic markers described in the current study can be utilized in breeding programs to select germplasm containing multiple superior alleles for spikelet number. This knowledge could also be applied in genomic selection to help improve the rate of genetic gain for spikelet number (Meuwissen et al. 2001; Goddard and Hayes 2007). Genomic prediction accuracy for spikelet number has been reported to be as high as 78% (Alqudah et al. 2020), but could potentially be improved further by including polymorphisms of known effect in the prediction model (Bernardo 2014; Rutkoski et al. 2014; Li et al. 2019). In simulations, marker effects with an *R*^2^ greater than 10% had a positive or neutral effect on prediction accuracy (Bernardo 2014). Therefore, including *WAPO-A1* (%*R*^2^ = 18.7; Table S6) and *QSn.csu-6B* (%*R*^2^ = 13.8; Table S6) loci as fixed effects could improve the accuracy of prediction models for spikelet number.

### Genetic constraints to regionally adapted germplasm

The positive correlation between spikelet number and heading date (Table S4) presents a constraint on selecting for increased spikelet number where regional adaptation favors an earlier heading date (Zheng et al. 2013; Kamran et al. 2014). This is reflected in the higher frequency of *Vrn-D3b* and *Ppd-B1b* alleles in wheat germplasm adapted to northern latitudes compared to southern latitudes (Fig. 5), likely because wheat varieties with later heading dates have a lower risk of cold damage from late-spring frost events (Kamran et al. 2014). Likewise, varieties that head earlier are likely to be well adapted to southern latitudes, where summer heat stress can negatively impact fertility. Therefore, genetic variation for spikelet number that does not impact heading date may be less regionally constrained, and has broader utility in breeding programs. Natural allelic variation at *WAPO-A1* had no significant effect on heading date in the COP-RIL population in Fort Collins 2020 (Table S8), and the frequencies of the superior *Wapo-A1b* allele in the HWWAMP were consistent between latitudes, suggesting there are fewer regional constraints to the use of this allele in the Great Plains. However, previous findings from a diversity panel of 518 European winter wheat varieties across three environments revealed that *WAPO-A1* has a mild, but significant effect on heading date (Muqaddasi et al. 2019), so additional studies in a more diverse set of environments will be required to fully characterize this allele.

Although only based on one year of field data, analysis of the HIFs and COP-RILs suggests that the *QSn.csu-6B* locus has no significant effect on heading date (Tables S2 and S8). Despite this observation, the *QSn.csu-6Bb* allele was detected in increasing frequency from southern to northern latitudes in the Great Plains, following the trend of the *Vrn-D3b* and *Ppd-B1b* allelic frequencies (Fig. 5). The *QSn.csu-6B* locus exhibits a significant two-way interaction with *VRN-D3* (Table S7), whereby the effect size of the *QSn.csu-6Bb* allele is greatest in germplasm carrying the early-heading *Vrn-D3a* allele (Fig. 3C). This suggests that the most promising breeding application of this allele may be in early-heading germplasm adapted to southern latitudes where the frequency of the *Vrn-D3a* allele is highest.

### Cloning the causative genetic variant underlying the *QSn.csu-6B* locus

Although hundreds of QTL associated with wheat yield components have been detected (Cao et al. 2020), few causative genes have been cloned. A powerful approach to identify and characterize the genetic variant underlying the *QSn.csu-6B* locus will be to perform high-resolution genetic mapping with near isogenic lines containing recombination breakpoints within the *QSn.csu-6B* region of interest. This effort could be accelerated by screening heterozygous individuals derived from the F_5_ HIFs described in this study, which, because of their homogeneous genetic backgrounds, provide an increased statistical power to detect small effect variants (Haley et al. 1994; Tuinstra et al. 1997). The use of exome capture sequencing (Krasileva et al. 2017) will help identify a high-density set of informative markers with which to precisely identify recombination breakpoints and delimit the QTL region.

Identification of the underlying causal gene will allow for the characterization of natural genetic variation within diverse panels of *Triticum* and *Triticum*-related species, for many of which genome and exome sequencing data is available (Krasileva et al. 2017; He et al. 2019). Furthermore, random and targeted mutagenesis can be applied to expand the range of genetic variation in both coding and regulatory regions, the value of which was recently demonstrated for agronomic traits in tomato (*Solanum lycopersicum* L.) (Rodríguez-Leal et al. 2017) and maize (*Zea mays*) (Liu et al. 2021). Screening this genetic diversity may reveal haplotypes and novel variants of utility for breeding programs.

Mutant alleles will also be valuable to further our understanding of the regulatory pathways determining spikelet number. These studies should include characterization of the complex three and four-way interactions between *VRN-D3*, *PPD-B1*, *WAPO-A1*, and *QSn.csu-6B* detected in this study (Fig. 3C; Table S7), potentially enabling more targeted approaches to achieving optimal heading date and spike architecture for target environments. An understanding of how these genetic loci interact with each other and their environment will be important to more efficiently breed wheat cultivars expressing high yield stability that are well adapted to specific target environments (Sreenivasulu and Schnurbusch 2012; Brinton and Uauy 2019).

## Conclusion

Variation at the *QSn.csu-6B* locus is consistently associated with spikelet number in hard winter wheat, exhibits epistatic interactions with three other alleles for spikelet number, and has only a limited impact on heading date. Despite these interactions, marker-assisted selection to combine superior alleles at these loci is a promising approach to increase spikelet number and help develop higher-yielding wheat varieties.

## Supplementary Information

Below is the link to the electronic supplementary material.

Supplementary file1 (PDF 497 KB)

Supplementary file2 (XLSX 17093 KB)

## Data Availability

All phenotypic and genotypic data for the hard winter wheat association mapping panel, biparental population, and heterogeneous inbred families used in this study are provided in supplemental data 1. This file also includes the calculated best linear unbiased estimates for the hard winter wheat association mapping panel and biparental population as well as the genome-wide associations calculated for kernel width for the hard winter wheat association mapping panel. Genetic materials used in this study are available from the authors upon request.
