# Supplementary material for: Identification and validation of a QTL for spikelet number on chromosome arm 6BL of common wheat (Triticum aestivum L.)
Source: Mol Breed. 2022 Mar 21;42(4):17. doi: 10.1007/s11032-022-01288-7 (PMC10248590; doi:10.1007/s11032-022-01288-7)

# Identification and validation of a QTL for spikelet number on chromosome arm 6BL of common wheat (*Triticum aestivum* L.)

## Supplemental file 1

**Table S1:** Genetic markers used in this study

| Marker | RefSeq v1.0 Pos | Locus | FAM primer (5'-3') | VIC primer (5'-3') | Common primer (5'-3') | Citation |
|---|---|---|---|---|---|---|
| KS0617_760354 | Chr6B:491,950,112 | *QSn.csu-6B* | tagctgttgtaggcgtgtga | tagctgttgtaggcgtgtgg | gcctaatgcgatttctgaattca | (Krasileva et al. 2017) |
| KS0617_760357 | Chr6B:492,023,835 | *QSn.csu-6B* | gcaaggtagtgatgataatcctcca | gcaaggtagtgatgataatcctccg | gctgctctttgataattttgcg | (Krasileva et al. 2017) |
| Bobwhite_c22638_135 | Chr6B:496,988,214 | *QSn.csu-6B* | aaactacgctcactagatgatgt | aaactacgctcactagatgatgc | ccaggtttgtggcaagatg | (Wang et al. 2014) |
| KS0617_760427 | Chr6B:502,486,272 | *QSn.csu-6B* | cattggaagagctgtgcacc | cattggaagagctgtgcact | tcaccgctgcatagtccg | (Krasileva et al. 2017) |
| 7AL_6666 | Chr7A:673,419,048 | *WAPO-A1* | gacaggaacgcgaacatggc | gacaggaacgcgaacatggg | cctcactcttccacgctgat | (Kuzay et al. 2019) |
| VRN-D3 | Chr7D:68,416,693 | *VRN-D3* | gccctcgtccgaccatggga | ccctcgtccgaccatgggg | gaagagcacgagcacgaagcgat | (Chen et al. 2010) |
| TaPpdBJ003 | Chr2B:56,230,452 | *PPD-B1* | N/A | cgtgaagagctagcgatgaaca | tgggcacgttaacacaccttt | (Beales et al. 2007) |

**Table S2:** Association between *QSn.csu-6B* and spikelet number in eight HIFs in the Fort Collins 2020 environment. Each HIF is fixed for a single allele at the *WAPO-A1*, *VRN-D3*, and *PPD-B1* loci and segregating for at the *QSn.csu-6B* locus. There was no significant marker-trait association between *QSn.csu-6B* and heading date.

| **Family** | **Mean spikelet number** | **Effect size (spikelets)** | ***P*-value** | **Heading Date (Julian Days)** | **Heading Date (C.I. 95%)** | ***WAPO-A1*** | ***VRN-D3*** | ***PPD-B1*** |
|---|---|---|---|---|---|---|---|---|
| **COP6BHF361** | 20.2 | 0.352 | 0.0053 | 149.7 | (149.6, 149.7) | *b* | *b* | *b* |
| **COP 6BHF260** | 19.8 | 0.504 | 0.0006 | 149.3 | (149.3, 149.4) | *a* | *b* | *b* |
| **COP 6BHF146** | 19.7 | 0.352 | 0.0041 | 149.8 | (149.7, 149.9) | *a* | *a* | *b* |
| **COP 6BHF347** | 19.1 | 0.370 | 0.0012 | 149.7 | (149.6, 149.7) | *b* | *b* | *b* |
| **COP 6BHF337** | 19.1 | 0.200 | 0.0667 | 146.2 | (146.1, 146.2) | *b* | *a* | *a* |
| **COP 6BHF275** | 19.0 | 0.248 | 0.0215 | 148.2 | (148.1, 148.2) | *b* | *a* | *b* |
| **COP 6BHF365** | 19.0 | 0.344 | 0.0013 | 148.3 | (148.3, 148.4) | *a* | *b* | *a* |
| **COP 6BHF207** | 18.1 | 0.441 | <0.0001 | 149.3 | (149.3, 149.4) | *a* | *a* | *b* |

**Table S3.** The log10 (*P*) values for the peak markers identified from the 90K SNP assay (Wang et al. 2014). The genome-wide associations were performed using 298 genotypes from the HWWMAP and the BLUEs calculated for kernel width, spikelet number, thousand kernel weight, and heading date from the Greeley 2012 dryland, Greeley 2012 irrigated, Fort Collins 2013 dryland, and Fort Collins 2013 irrigated environments. Log10 (*P*) values for all traits and markers can be found in supplemental data 1.

| **Marker** | **RefSeq v1.0 Pos** | **Locus** | **Kernel width** | **Spikelet number** | **Thousand kernel weight** | **Heading date** |
|---|---|---|---|---|---|---|
| Bobwhite_c22638_135 | Chr6B:496,988,214 | *QSn.csu-6B* | 3.358257 | 0.255466 | 3.686106 | 0.090588 |
| IWA5913 | Chr7A:674,276,906 | *WAPO-A1* | 0.249613 | 7.642443 | 0.103143 | 0.748676 |
| BS00063966_51 | Chr7D:66,824,781 | *VRN-D3* | 0.181616 | 0.678392 | 0.003124 | 0.647817 |
| Kukri_c16479_765 | Chr2B:54,778,663 | *PPD-B1* | 0.676399 | 0.201718 | 0.183635 | 3.554004 |

**Table S4**. Correlations between heading date (HD), thousand kernel weight (TKW) and spikelet number (SNS) in the HWWAMP for four environments and calculated BLUEs across environments. Pearson's product moment correlation coefficient and *P*-value are shown. N = 299 for all environments and BLUEs.

| Environment | Trait | Correlation | *P*-value |
|---|---|---|---|
| Greeley 2012 Dry | TKW vs. SNS | -0.028 | 0.6340 |
| | TKW vs. HD | -0.343 | <0.0001 |
| | SNS vs. HD | -0.107 | 0.0651 |
| Greeley 2012 Irrigated | TKW vs. SNS | -0.408 | <0.0001 |
| | TKW vs. HD | -0.611 | <0.0001 |
| | SNS vs. HD | 0.465 | <0.0001 |
| Fort Collins 2013 Dry | TKW vs. SNS | -0.255 | <0.0001 |
| | TKW vs. HD | -0.188 | 0.0011 |
| | SNS vs. HD | 0.194 | 0.0007 |
| Fort Collins 2013 Irrigated | TKW vs. SNS | -0.207 | 0.0003 |
| | TKW vs. HD | -0.198 | 0.0006 |
| | SNS vs. HD | 0.336 | <0.0001 |
| BLUEs | TKW vs. SNS | -0.260 | <0.0001 |
| | TKW vs. HD | -0.347 | <0.0001 |
| | SNS vs. HD | 0.389 | <0.0001 |

**Table S5**: Correlation between spikelet number (SNS), grain length, grain width, and thousand kernel weight (TKW) for the COP-RIL population in the Fort Collins 2017 environment based on BLUEs. Values indicate Pearson's product moment correlation. * = *P* <0.05, *** = *P* <0.0001.

| BLUEs | Length | Width | TKW |
|---|---|---|---|
| **SNS** | -0.163* | -0.266*** | -0.184*** |
| **Length** | - | 0.454*** | 0.659*** |
| **Width** | - | - | 0.870*** |

**Table S6:** ANOVA *P*-values for the marker-trait association between the *QSn.csu-6B*, *WAPO-A1*, *Vrn-D3*, and *PPD-B1* loci and spikelet number in the COP-RIL population for Fort Collins 2017, Fort Collins 2019, Fort Collins 2020 and BLUEs.

| | | *QSn.csu-6B* | | | *WAPO-A1* | | | *VRN-D3* | | | *PPD-B1* | | |
|---|---|---|---|---|---|---|---|---|---|---|---|---|---|
| Environment | N | Effect size | *P*-value | %$R^2$ | Effect size | *P*-value | %$R^2$ | Effect size | *P*-value | %$R^2$ | Effect size | *P*-value | %$R^2$ |
| 2017 | 211 | 0.808 | <0.0001 | 12.9 | 0.921 | <0.0001 | 14.5 | 0.46 | 0.0003 | 7.1 | 0.422 | 0.0013 | 6.3 |
| 2019 | 209 | 0.709 | <0.0001 | 9.2 | 1.01 | <0.0001 | 17.1 | 0.518 | 0.0006 | 5.1 | 0.331 | 0.0371 | 1.6 |
| 2020 | 215 | 0.679 | <0.0001 | 11.6 | 0.713 | <0.0001 | 11.8 | 0.576 | <0.0001 | 8.9 | 0.42 | 0.0016 | 4.1 |
| BLUEs | 215 | 0.739 | <0.0001 | 13.8 | 0.89 | <0.0001 | 18.7 | 0.518 | <0.0001 | 7.1 | 0.394 | 0.0031 | 3.6 |

**Table S7.** Sum of squares and *P*-values for the interaction terms calculated from the COP-RIL population from the Fort Collins 2017, 2019, and 2020 environments via a Tukey-Adjusted Type-2 ANOVA.

| Term | Sum Sq. | P-value |
|---|---|---|
| *WAPO-A1* | 119.8 | <0.0001 |
| *QSn.csu-6B* | 83.25 | <0.0001 |
| *VRN-D3* | 49.53 | <0.0001 |
| *PPD-B1* | 18.94 | <0.0001 |
| Environment | 535.39 | <0.0001 |
| *WAPO-A1* : *QSn.csu-6B* | 2.87 | 0.086 |
| *WAPO-A1* : *VRN-D3* | 3.69 | 0.052 |
| *QSn.csu-6B* : *VRN-D3* | 10.85 | 0.0009 |
| *WAPO-A1* : *PPD-B1* | 0.41 | 0.519 |
| *QSn.csu-6B* : *PPD-B1* | 0.8 | 0.364 |
| *VRN-D3* : *PPD-B1* | 0.63 | 0.421 |
| *WAPO-A1* : *QSn.csu-6B* : *VRN-D3* | 0.29 | 0.589 |
| *WAPO-A1* : *QSn.csu-6B* : *PPD-B1* | 1.35 | 0.240 |
| *WAPO-A1* : *VRN-D3* : *PPD-B1* | 0 | 0.997 |
| *QSn.csu-6B* : *VRN-D3* : *PPD-B1* | 13.28 | 0.0002 |
| *WAPO-A1* : *QSn.csu-6B* : *VRN-D3* : *PPD-B1* | 15.26 | <0.0001 |

**Table S8:** Effect size and significance of spikelet number and heading date in the COP-RIL population from Fort Collins 2020.

| | Spikelet number | | Heading date | |
|---|---|---|---|---|
| Locus | *P*-value | Effect size (spikelets) | *P*-value | Effect size (days) |
| *QSn.csu-6B* | <0.0001 | 0.679 | 0.107 | 0.326 |
| *WAPO-A1* | <0.0001 | 0.713 | 0.95 | 0.0135 |
| *VRN-D3* | <0.0001 | 0.576 | <0.0001 | 1.31 |
| *PPD-B1* | 0.001 | 0.420 | <0.0001 | 1.16 |

**Table S9:** The frequency of individual lines containing pairs of positive effect alleles in the COP-RIL population (N = 215, $X^2$ = 33.80, *P*-value < 0.001).

| | ***VRN-D3b*** | ***PPD-B1b*** | ***WAPO-A1b*** |
|---|---|---|---|
| ***QSn.csu-6Bb*** | 0.31 | 0.23 | 0.21 |
| ***VRN-D3b*** | - | 0.18 | 0.13 |
| ***PPD-B1b*** | - | - | 0.13 |

**Figure S1:** Tree diagram representing spikelet number for allelic combinations of *PPD-B1*, *WAPO-A1*, *VRN-D3*, and *QSn.csu-6B* in the COP-RIL population. Each node of the tree includes mean spikelets per spike ± the standard error. Significant pairwise comparisons between allelic pairs are denoted by * = *P* <0.05.

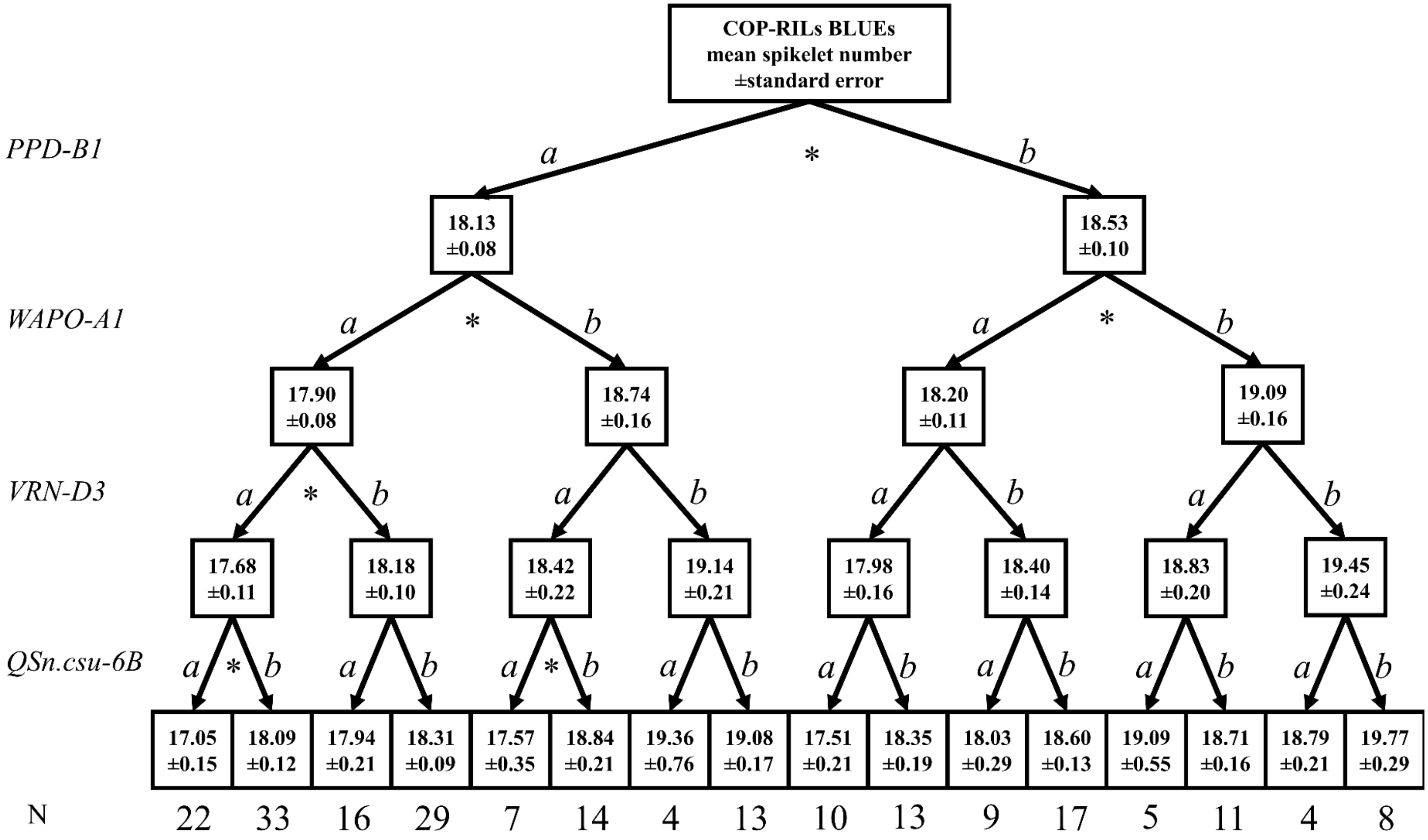

**Figure S2.** Mean spikelet number by heading date for the COP-RIL population in the Fort Collins 2020 environment. The X-axis is heading date in Julian Days, Y-axis is mean spikelet number and each line is represented by a color point denoting the allele present. Spikelet number and heading date are positively correlated (r = 0.37, CI95 = 0.25-0.48, $P < 0.0001$). Each scatter plot represents a linear regression for each allele of a given loci of interest and the shaded area is the 95 % confidence level interval of the regression

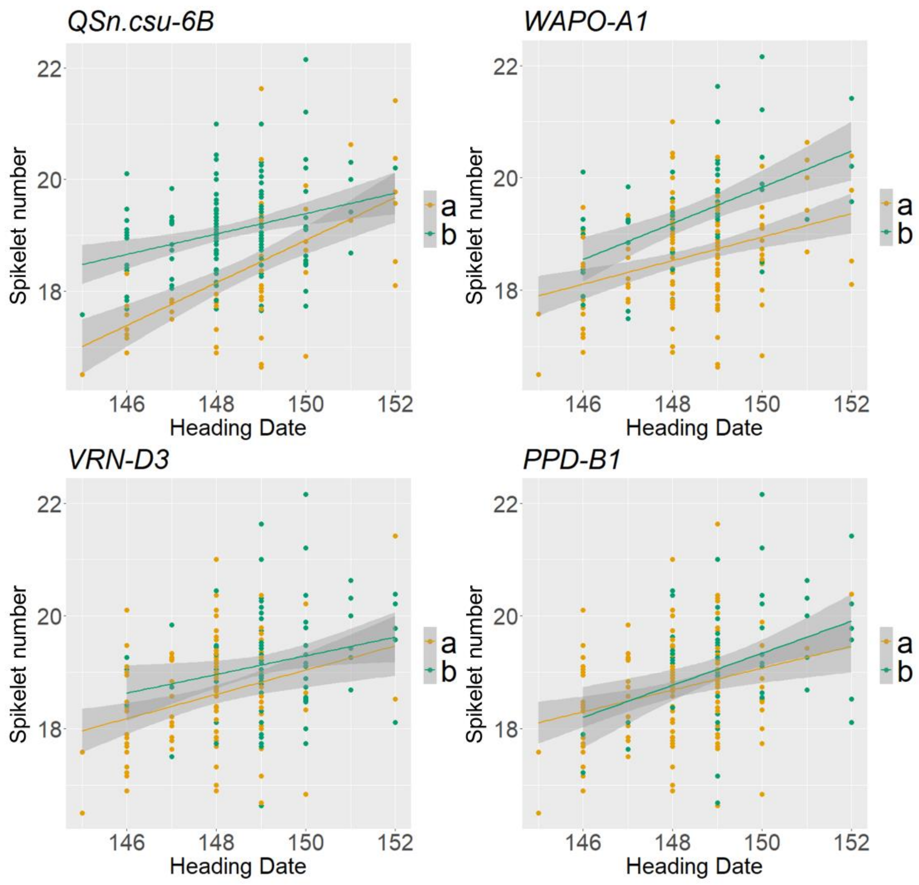

Supplement: Supplementary file 1 — Supplementary file1 (PDF 497 KB) [file 11032_2022_1288_MOESM1_ESM.pdf]
